# TMT-Based Quantitative Proteomics Reveals the Molecular Mechanism Underlying Quality Deterioration of Refrigerated Northern Pike (*Esox lucius*)

**DOI:** 10.3390/foods15173088

**Published:** 2026-08-31

**Authors:** Tianyu Hou, Rui Ma, Weiqun Wang, Shijie Bi, Gao Gong

**Affiliations:** 1 College of Food Science and Pharmacy, Xinjiang Agricultural University, Urumqi 830052, China; hty20040929@163.com (T.H.); marui250609@163.com (R.M.); 18259359458@163.com (W.W.); bsj19941001@163.com (S.B.); 2 College of Animal Sciences, Xinjiang Agricultural University, Urumqi 830052, China

**Keywords:** *Esox lucius*, proteomics, low-temperature storage, quality changes

## Abstract

This study aimed to elucidate the characteristics of protein changes and their relationship with physicochemical properties during the quality deterioration of refrigerated Northern Pike (*Esox lucius*). We employed tandem mass tag (TMT)-based quantitative proteomics, combined with physicochemical indicators, including surface hydrophobicity, carbonyl content, sulfhydryl content, and myofibrillar fragmentation index (MFI), to systematically analyze protein alterations during cold storage. The results showed that with prolonged cold storage, the surface hydrophobicity, carbonyl content, and MFI of Northern Pike samples exhibited an increasing trend, whereas the sulfhydryl content decreased. Proteomic analysis identified a total of 563 differentially expressed proteins (DEPs) between fresh and cold-stored states, of which 293 were upregulated and 270 were downregulated. Furthermore, by integrating COG/KOG functional classification, subcellular localization, and GO enrichment analysis, the functional characteristics of the DEPs and their potential involvement in biological processes were systematically characterized. Correlation analysis revealed that six DEPs were significantly correlated with the changes in physicochemical indices of Northern Pike during cold storage, namely fructose-bisphosphate aldolase encoded by ALDOA, Nesh-SH3 domain-target protein, keratin, neurofilament heavy polypeptide, serine/arginine repetitive matrix protein 2-like, and asymmetrical bis(5′-nucleosidyl) tetraphosphatase. By integrating analyses of differentially abundant proteins and protein oxidation, this study suggests potential molecular mechanisms potentially responsible for quality loss in northern pike under chilled storage, offering a theoretical foundation for its preservation and quality assessment.

## 1. Introduction

The northern pike (*Esox lucius*) is a large, cold-water, carnivorous fish that primarily inhabits the cold-water basins of the Northern Hemisphere [1]. In China, it is mainly distributed in the Irtysh River Basin in the Altay region of Xinjiang. This species exhibits fast growth, high nutritional value, and a desirable taste after cooking. As a commercially important fish in Xinjiang, it has promising development prospects. However, owing to its limited geographical distribution and regional constraints, northern pike often requires prolonged storage and transportation from its place of origin to consumer markets, during which its quality is highly susceptible to deterioration [2]. This not only restricts the market circulation of the product but also impedes the further development of the industry.

Low-temperature preservation is widely applied in the aquatic product sector [3]. Among the available methods, refrigerated storage, typically at temperatures within the range of 0–4 °C, can effectively inhibit microbial growth and reproduction, reduce endogenous enzyme activity in fish, and decelerate biochemical reaction rates, such as lipid oxidation and protein degradation [4], thereby delaying spoilage and extending shelf life. For northern pike, which is prone to quality loss during storage and transportation, cold storage can, to a certain extent, mitigate water loss and quality decline while preserving acceptable eating quality [5]. Nevertheless, with prolonged cold storage, muscle proteins inevitably undergo oxidation, denaturation, and degradation [6], which compromises flesh quality. Currently, compared with livestock and poultry meat, studies on the mechanisms of muscle protein changes in high-value cold-water fish, such as *Esox lucius*, during refrigerated storage remain insufficient [7]. The patterns of alteration in the protein structure and functional properties of northern pike under refrigerated conditions, along with their relationship to quality deterioration, have yet to be systematically elucidated.

Proteomics focuses on the proteome to systematically investigate the composition and dynamic changes in intracellular proteins [8]. Proteomic technologies have been employed in freshwater fish research [9,10,11]. In particular, quantitative proteomics approaches, represented by tandem mass tag (TMT) and isobaric tags for relative and absolute quantitation (iTRAQ), are increasingly utilized owing to their high throughput, reproducibility, accuracy, and low quantification error [12]. TMT allows for multiplexed analysis of up to 10 samples simultaneously, which minimizes inter-batch variability and ensures more accurate quantitative comparisons across samples. In contrast, iTRAQ, although similar in principle, has a lower multiplexing capacity and reduced sensitivity than TMT [13]. Although quantitative proteomics has made considerable advances in investigating quality changes in freshwater fish, the application of an integrated strategycombining TMT-based quantitative proteomics with Spearman correlation analysis to identify proteins potentially associated with quality deterioration in cold-water fish remains insufficiently explored. In this study, we measured physicochemical indicators, including surface hydrophobicity, carbonyl content, sulfhydryl content, and myofibrillar fragmentation index (MFI), in northern pike fillets at multiple time points during cold storage. We also applied TMT-based quantitative proteomics to compare the protein profiles of fresh samples (day 0) and samples stored in the refrigerator for 15 d. By further integrating Gene Ontology (GO) functional annotation, Clusters of Orthologous Groups/Eukaryotic Orthologous Groups (COG/KOG) functional classification, and correlation analyses between differentially expressed proteins and physicochemical indicators, the influence of cold storage conditions on northern pike quality was systematically explored in this study. The findings are expected to provide a reference for the development of cold storage preservation technology for northern pike and for research on the quality deterioration patterns of freshwater fish.

## 2. Materials and Methods

### 2.1. Raw Material and Sampling

Live fresh northern pikes (*Esox lucius*) of similar size were purchased from a local market and transported to the laboratory. After measuring body weight and body length, the fish were euthanized by a blow to the head, and the head, viscera, and skin were removed. The muscle was washed with pre-chilled water, blotted dry with filter paper, and the dorsal muscle was cut into fillets measuring 2 cm × 1 cm × 1 cm. A portion of the fillets was immediately analyzed as a day 0 sample. The remaining fillets were packed in polyethylene bags, stored at 4 °C, and sampled on days 1, 3, 5, 7, 10, and 15 to measure the relevant indices. For TMT-based quantitative proteomics, muscle samples collected on days 0 and 15 were designated as the control and cold storage groups, respectively.

### 2.2. Analysis of Physicochemical Indicators

#### 2.2.1. Extraction of Myofibrillar Proteins

Myofibrillar proteins were isolated as described by Niu et al. [14] with minor modifications. Briefly, minced muscle (2 g) was blended with 20 mL of isolation buffer (10 mM potassium phosphate, pH 7.0, 0.1 M NaCl, 2 mM MgCl_2_, and 1 mM EDTA) at 7000 rpm for 30 s. After centrifugation (6000 rpm, 10 min, 4 °C), the supernatant was removed. This extraction step was repeated three times with the pellet. The pellet was subsequently rinsed thrice with 0.1 M NaCl. Each rinse involved resuspending the pellet, centrifuging under the same conditions, and discarding the supernatant. In the final wash, the mixture was strained through a four-layer gauze, and the filtrate was retained as the myofibrillar protein preparation. Protein content was measured using the BCA assay and then diluted to 2 mg/mL. The preparation was stored at 4 °C and analyzed within 24 h.

#### 2.2.2. Determination of Surface Hydrophobicity

Surface hydrophobicity was assessed using a modified protocol based on that of Mitra et al. [15]. A 1 mL volume of myofibrillar protein solution (2 mg/mL) was combined with 200 µL of bromophenol blue (BPB; 1 mg/mL) and mixed vigorously. For the blank, the protein solution was replaced with a 10 mM potassium phosphate buffer (0.6 M KCl, pH 7.4). After holding at 25 °C for 10 min, the sample was centrifuged (3000 rpm, 15 min), the resulting supernatant was diluted tenfold, and its absorbance was read at 595 nm. The quantity of bound BPB was determined using Equation (1), and all analyses were performed in triplicate.

(1) $$\mathrm{Bound}\;\mathrm{BPB}\;(\mu g)=\frac{A_{0}-A_{1}}{A_{0}}\;\times \;200$$

where A_0_ and A_1_ represent the absorbance of the blank and the sample, respectively.

#### 2.2.3. Determination of Carbonyl Content

Carbonyl content was measured following a modified version of the protocol reported by Gong et al. [16]. Briefly, a 1 mL aliquot of myofibrillar protein solution (2 mg/mL) was combined with 1 mL of 10 mM DNPH (prepared in 2 M HCl). The reaction was allowed to proceed at room temperature in the dark for 60 min with vortex mixing at 15-min intervals. For the blank control, the DNPH solution was replaced with 2 M HCl. After incubation, 1 mL of 20% TCA was added, and the sample was centrifuged (10,000× *g*, 5 min). The collected precipitate was rinsed three times with 1 mL of ethanol/ethyl acetate (1:1, *v*/*v*) to eliminate residual DNPH. The washed pellet was then dissolved in 3 mL of 6 M guanidine hydrochloride (in 2 M HCl) and heated at 37 °C for 15 min to solubilize the proteins. Following centrifugation (10,000× *g*, 5 min, 4 °C), the absorbance of the supernatant was measured at 370 nm. The carbonyl content was computed using Equation (2), and all determinations were performed in triplicate.

(2) $$\mathrm{Carbonyl}\;\mathrm{content}\;(\mathrm{nmol}/\mathrm{mg}\;\mathrm{prot})\;=\frac{\left(A_{1}-A_{2}\right)\;\times \;3\;\times {\;10}^{6}}{\varepsilon \;\times c}$$

where A_1_ is the absorbance of the sample at 370 nm, A_2_ is the absorbance of the blank at 370 nm, 3 is the dilution factor, ε is the molar extinction coefficient (22,000 L/(mol∙cm)), and c is the myofibrillar protein concentration (mg/mL).

#### 2.2.4. Determination of Sulfhydryl Content

The sulfhydryl content of northern pike myofibrillar proteins was measured using a Total Sulfhydryl Content Assay Kit (Beijing Solarbio Science & Technology Co., Ltd., Beijing, China).

#### 2.2.5. Determination of Myofibrillar Fragmentation Index (MFI)

The myofibrillar fragmentation index (MFI) was determined using a protocol described by Fu et al. [17], with minor modifications. Minced muscle (2 g) was homogenized at 7000 rpm for 1 min in 10 volumes of ice-cold MFI buffer (100 mM KCl, 11.2 mM K_2_HPO_4_, 8.8 mM KH_2_PO_4_, 1 mM EDTA, and 1 mM MgCl_2_). After centrifugation (4000× *g*, 10 min, 4 °C), the supernatant was removed. The pellet was washed twice by resuspension in the same buffer, followed by centrifugation. The final pellet was resuspended in 10 mL of MFI buffer and strained through a 74 µm nylon mesh to eliminate the connective tissue. The protein concentration of the filtrate was determined using a BCA assay kit and adjusted to 0.5 ± 0.05 mg/mL. Absorbance was recorded at 540 nm, and the MFI was calculated using Equation (3), based on triplicate measurements.

MFI = A_540_ × 200 (3)

where A_540_ is the absorbance of the myofibrillar solution at 540 nm wavelength.

### 2.3. Statistical Analysis

Data are expressed as the mean ± standard deviation to ensure reliability. Statistical analyses were performed using SPSS 27.0 software. One-way analysis of variance (ANOVA) was used to evaluate the significant differences. When ANOVA indicated a significant overall effect, multiple comparisons were conducted using Duncan’s test, with a significance level set at *p* < 0.05. Graphical plots were generated using the Origin 2024 software.

### 2.4. DTMT Quantitative Proteomics

#### 2.4.1. Protein Extraction

Northern pike muscle tissue was homogenized in ice-cold RIPA buffer (25 mM Tris-HCl, pH 7.6, 15 mM NaCl, 1% NP-40, 1% sodium deoxycholate, 1% SDS, and protease inhibitor cocktail) at a 1:10 (*w*/*v*) ratio. Homogenization involved low-temperature grinding for 4 min, followed by sonication in an ice-water bath for 20 min. After centrifugation (12,000 rpm, 10 min, 4 °C), the supernatant was collected. Protein concentration was measured using the BCA assay with BSA as the reference standard, and the absorbance was read at 562 nm. A 100 μg aliquot of total protein was mixed with five volumes of acetone (pre-cooled to −20 °C) and stored overnight at −20 °C to precipitate the proteins. The sample was then centrifuged (12,000 rpm, 10 min, 4 °C), the supernatant discarded, and the pellet washed twice with 200 μL of ice-cold 80% acetone. Following an additional centrifugation step under identical conditions, the supernatant was removed and the resulting protein pellet was collected.

#### 2.4.2. Trypsin Digestion and Peptide Labeling

Protein pellets were first reconstituted in 100 μL of reconstitution buffer by vortexing and sonication (3 min). Disulfide reduction was performed by adding dithiothreitol (DTT) to 5 mM and incubating at 55 °C with agitation for 20 min. After the mixture was cooled to room temperature, alkylation was performed with 15 mM iodoacetamide (IAA) in the dark for 30 min. Sequencing-grade trypsin was introduced at an enzyme-to-substrate ratio of 1:50 (*w*/*w*), and digestion was allowed to proceed overnight at 37 °C with shaking. The resulting peptides were labeled with a TMT reagent kit (Thermo Scientific, Rockford, IL, USA) following the manufacturer’s protocol, after which equal amounts of the labeled samples from each group were combined for analysis. To remove sodium deoxycholate (SDC), trifluoroacetic acid (TFA) was added to a final concentration of 2% to induce its precipitation. After thorough mixing, the sample was centrifuged (12,000 rpm, 10 min, 4 °C), and the supernatant was retained. The precipitate was re-extracted twice with 2% TFA, and all supernatants were pooled and subjected to another centrifugation step (12,000 rpm, 10 min, 4 °C) to obtain the labeled peptide mixture. Finally, the pooled peptides were desalted on a C18 solid-phase extraction (SPE) cartridge, and the eluate was dried by vacuum centrifugation overnight at 4 °C. The TMT 10-plex Isobaric Label Reagent Set (Thermo Scientific) was used for peptide labeling, with the following reporter ion channel assignments: channels 126, 130N, and 131N for the control groups and channels 127C, 128C, and 129C for the cold storage groups.

#### 2.4.3. Reversed-Phase Chromatographic Separation

Labeled peptides were resolved using liquid chromatography on an XBridge BEH C18 XP column. Solvent A was 10 mM ammonium acetate in water (pH 10), and solvent B was 10 mM ammonium acetate in acetonitrile/water (9:1, *v*/*v*; pH 10). The flow rate was maintained at 0.3 mL/min. A 60-min linear gradient was applied as follows: 5% B for 2 min, 5–30% B for 40 min, 30–40% B for 10 min, 40–90% B for 4 min, hold at 90% B for 2 min, and return to 2% B in 2 min. Fractions were collected every 1 min, pooled into 12 combined fractions, vacuum-dried, and stored at −80 °C.

#### 2.4.4. LC–MS/MS Analysis

Peptide samples (2 μL per injection) were introduced into an EASY-nLC 1200 UPLC system and resolved on a reverse-phase C18-AQ column (15 cm × 100 μm I.D., 1.9 μm particles; Reprosil-Pur 120) at a constant flow of 300 nL/min. The column outlet was directly interfaced via a nanoelectrospray ion source with a Q Exactive HFX mass spectrometer. The mobile phases consisted of 0.1% formic acid in water/acetonitrile (98:2, *v*/*v*; solvent A) and 0.1% formic acid in acetonitrile/water (80:20, *v*/*v*; solvent B). After column equilibration in 100% A, the autosampler directly injected the samples, and a 120-min gradient was applied at 300 nL/min. Positive ion data-dependent acquisition (DDA) was employed throughout the 120-min analysis. Survey full scans (*m*/*z* 350–1600) were collected at 120,000 resolution with an AGC target of 3 × 10^6^ and a maximum injection time of 30 ms. The 20 most abundant precursors per scan were isolated (quadrupole isolation window 0.7 *m*/*z*) and fragmented via HCD at a normalized collision energy of 32%. Fragment ion spectra were recorded at 45,000 resolution (fixed first mass 110 *m*/*z*), using an AGC target of 1 × 10^5^ and a maximum injection time of 96 ms. Dynamic exclusion was set to 45 s (based on chromatographic peak width), and precursor ions with charge states of 1+ or >6+ were excluded from MS/MS. Data acquisition was performed using default instrument settings without additional co-isolation/interference filtering criteria applied during acquisition. The Q Exactive HFX was operated in data-dependent acquisition (DDA) mode with HCD fragmentation at a normalized collision energy of 32%, as previously described.

#### 2.4.5. Bioinformatics Analysis

Raw MS data were processed using SpectroMine software (v4.2.230428.52329; Biognosys AG, Schlieren, Switzerland) with a built-in Pulsar search engine. MS/MS spectra were searched against the UniProt *Esox lucius* database (uniprotkb_Esox_lucius_2024_08_28.fasta). Trypsin was specified as the protease, allowing up to two missed cleavages. Carbamidomethylation of cysteine (+57.021 Da) and TMT10plex labels on lysine and N-termini were set as fixed modifications, whereas oxidation of methionine (+15.995 Da) and acetylation of protein N-termini (+42.011 Da) were set as variable modifications. The precursor and fragment mass tolerances were both 20 ppm. Peptide spectrum match and peptide identifications were filtered at a false discovery rate ≤ 1%. For protein quantification, TMT reporter ion intensities were extracted, corrected for isotopic impurities using the manufacturer-provided correction matrix, and normalized using the median method across all samples. Only unique and razor peptides were used for protein-level quantification.

After FDR filtering, proteins detected in all samples with at least one unique peptide were retained [18]. Quantitative data were log-transformed and centered using R (version 3.6.3) prior to subsequent analyses. Differentially expressed proteins were defined by a fold change ≥1.2 or ≤0.83 and a *t*-test *p*-value < 0.05 [19]. Volcano plots were generated with R (v3.3.2), and hierarchical clustering was performed and displayed as heatmaps using R (v1.0.12). For COG/KOG functional classification, protein sequences of the differentially abundant proteins were searched against the eggNOG 5.0 database using NCBI BLAST+ (v2.12.0), and the resulting functional annotations were summarized and visualized with R (version 3.3.2). Subcellular localization was predicted using CELLO (v2.5) and LOTREC3 (v1.0.1), and the distribution of subcellular localizations was summarized and visualized with R (version 3.3.2). For functional annotation, Gene Ontology (GO) analysis was performed using the GO database (release 2023.07.12) via Blast2GO (v5.2), with terms classified into biological process, cellular component, and molecular function categories.

### 2.5. Spearman’s Rank Correlation Analysis

Spearman’s rank correlation analysis was performed based on the relative abundances of the 563 DAPs and the measured values of the physicochemical indicators of northern pike fillets stored under cold conditions. For each protein–indicator pair, we computed the correlation coefficient (ρ) and the associated *p*-value, and corrected these *p*-values for multiple tests using the Benjamini–Hochberg method. Pairs meeting the corrected *p*-value < 0.05 and |ρ| > 0.8 thresholds were retained as satisfying both statistical significance and effect size requirements. The identified DAPs that met these criteria were used for heatmap visualization and further interpretation.

## 3. Results

### 3.1. Surface Hydrophobicity

**Figure 1:**
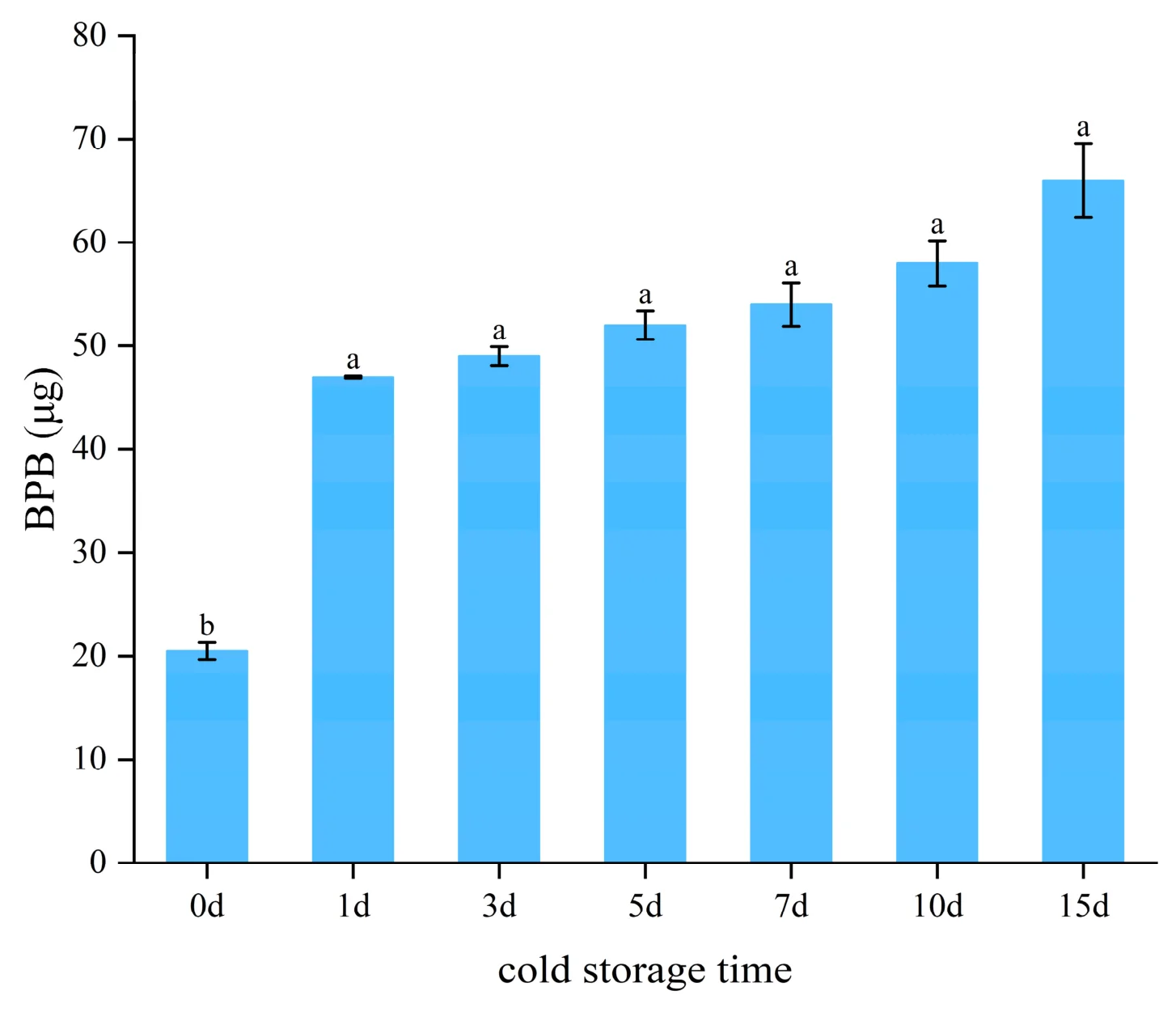
Changes in surface hydrophobicity of *Esox lucius* muscle proteins during refrigerated storage at 4 °C for 0, 1, 3, 5, 7, 10, and 15 days. Surface hydrophobicity was determined by measuring the bromophenol blue (BPB) binding capacity. Data are presented as mean ± SD (*n* = 3 biologically independent samples per time point). Statistical analysis was performed using one-way ANOVA followed by Tukey’s post hoc test, with significant differences between time points denoted by different letters (*p* < 0.05). BPB: bromophenol blue; SD: standard deviation; ANOVA: analysis of variance.

Surface hydrophobicity indicates the degree of protein denaturation [20]. Higher surface hydrophobicity corresponds to a greater amount of bound bromophenol blue (BPB) dye. As shown in Figure 1, the bound BPB of northern pike increased sharply during the first 0–1 days of cold storage; thereafter, the rate of increase slowed, yet an overall upward trend persisted, reaching 66.0 ± 3.59 μg on day 15. This behavior can be attributed to protein denaturation upon exposure to cold storage conditions, wherein the spatial structure of the protein is disrupted. In the early stages of storage, exposure of the internal hydrophobic groups causes a rapid increase in surface hydrophobicity. As storage time progresses, the degree of protein denaturation intensifies, and additional hydrophobic groups are gradually exposed, resulting in a further but decelerated increase in surface hydrophobicity. This trend is consistent with that of the control group reported by Lin et al. [21] on the effect of phytic acid treatment on the surface hydrophobicity of refrigerated yellowfin seabream (*Acanthopagrus latus*) muscle.

### 3.2. Carbonyl Content

**Figure 2:**
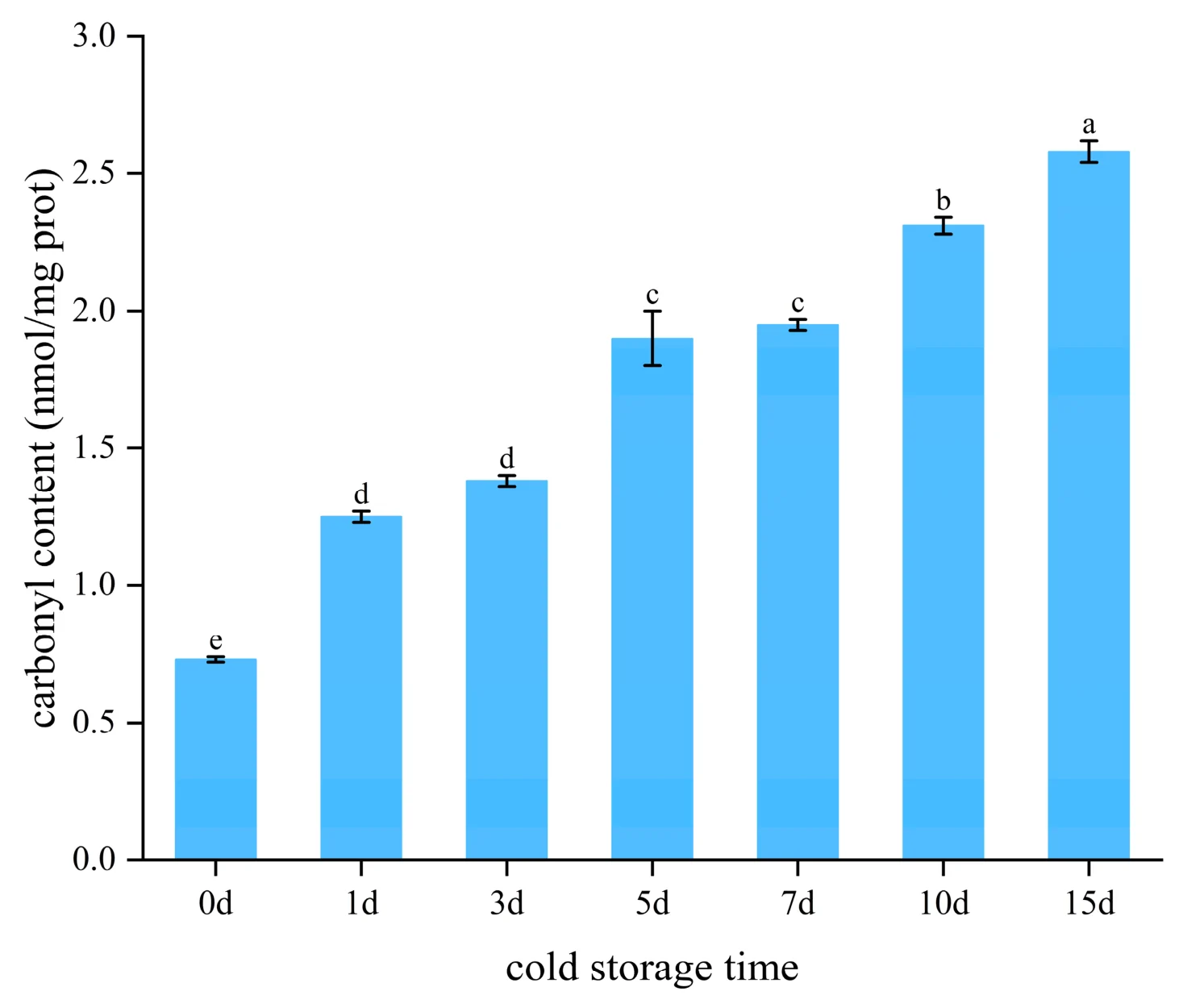
Changes in carbonyl content of *Esox lucius* muscle proteins during refrigerated storage at 4 °C for 0, 1, 3, 5, 7, 10, and 15 days. Data are presented as mean ± SD (*n* = 3 biologically independent samples per time point). Statistical analysis was performed using one-way ANOVA followed by Tukey’s post hoc test, with significant differences between time points denoted by different letters (*p* < 0.05). SD: standard deviation; ANOVA: analysis of variance.

Carbonyl group generation is one of the most prominent hallmarks of protein oxidation; therefore, carbonyl content serves as a key index for evaluating the degree of oxidation [22]. As illustrated in Figure 2, the carbonyl content of northern pike under refrigeration displayed a persistent upward trend from day 0 to day 15, increasing from 0.73 ± 0.01 nmol/mg prot on day 0 to 2.58 ± 0.04 nmol/mg prot on day 15. Specifically, the carbonyl content rose sharply from day 0 to day 5, remained relatively stable from day 5 to day 7, and then increased continuously from day 7 to day 15. During cold storage, the structural stability of proteins declines, rendering amino acid side chains more susceptible to reactions with reactive oxygen species, generating carbonyls. As storage time increases, the progressive accumulation of lipid oxidation products and the impact of microbial metabolism further intensify oxidative reactions, thereby driving a sustained elevation in carbonyl content [23]. This pattern is consistent with that observed in the control group of grass carp in the study by Shan et al. [24] on the mechanisms of transport stress-mediated muscle quality deterioration in refrigerated fish. These results demonstrate that protein oxidation in northern pike occurs continuously throughout the refrigerated storage period.

### 3.3. Sulfhydryl Content

**Figure 3:**
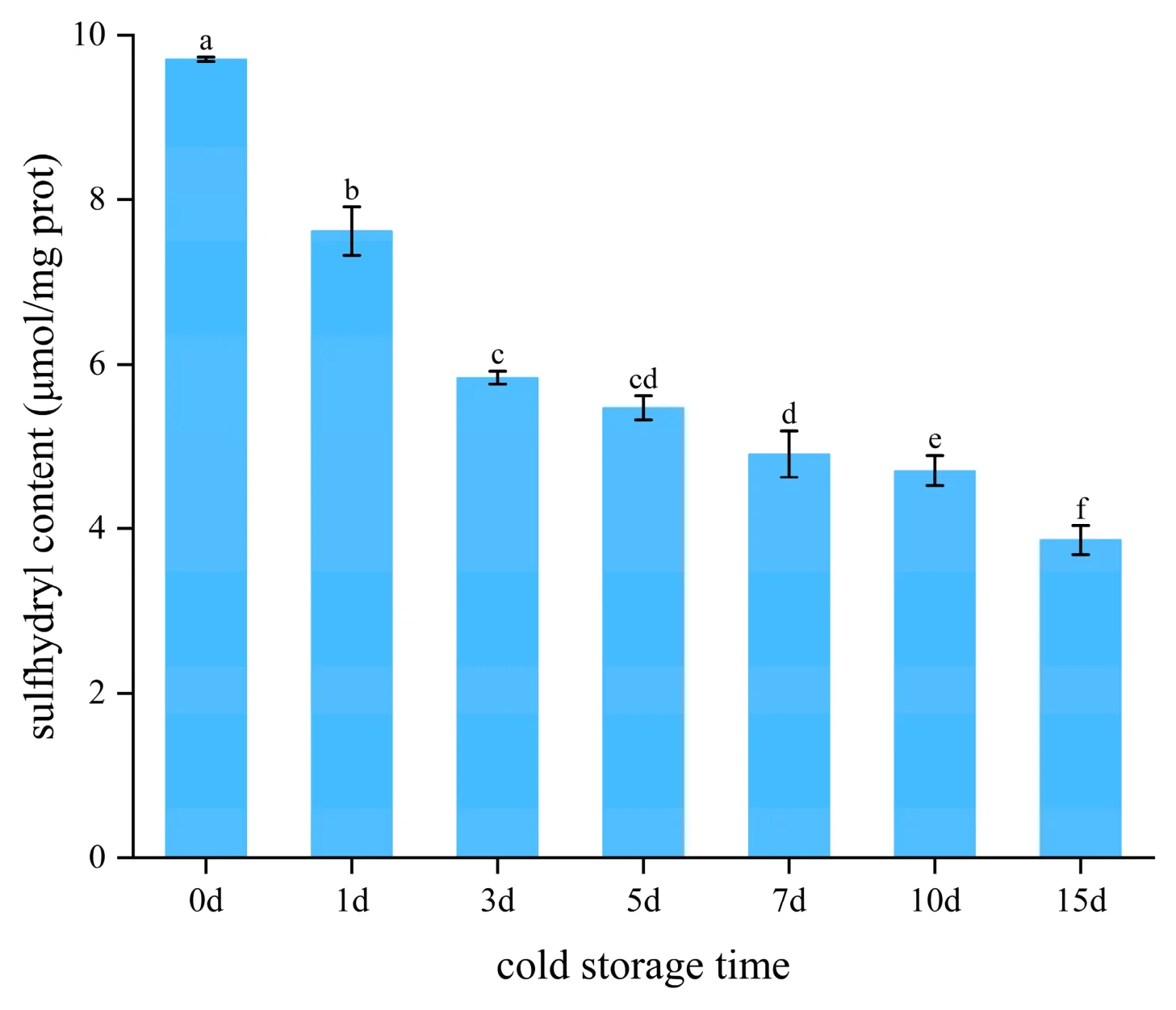
Changes in sulfhydryl content of *Esox lucius* muscle proteins during refrigerated storage at 4 °C for 0, 1, 3, 5, 7, 10, and 15 days. Data are presented as mean ± SD (*n* = 3 biologically independent samples per time point). Statistical analysis was performed using one-way ANOVA followed by Tukey’s post hoc test, with significant differences between time points denoted by different letters (*p* < 0.05). SD: standard deviation; ANOVA: analysis of variance.

Myofibrillar protein sulfhydryl groups are prone to oxidation, resulting in intramolecular or intermolecular cross-links and disulfide bonds. The decline in sulfhydryl content during frozen storage is primarily due to disulfide bond formation [25]. Therefore, monitoring changes in sulfhydryl content can serve as an indicator of protein oxidation during refrigerated storage of northern pike. As shown in Figure 3, the sulfhydryl content declined continuously from day 0 to day 15, with a decrease of 60.25% compared to the initial level by day 15. This declining trend aligns with the sulfhydryl content changes observed in Basa fish during frozen storage, as reported by Sriket et al. [26]. The results indicate that disulfide bonds are progressively formed in myofibrillar proteins, causing a sustained reduction in sulfhydryl content, a pattern consistent with the concurrent increase in carbonyl content, further confirming that continuous protein oxidation occurs in northern pike under cold storage conditions. A complete list of all quantified proteins, including accession numbers, gene names, unique peptide counts, fold changes, *p*-values, Q-values, and abundance values (both normalized and original intensities) for all quantified proteins, is provided in Supplementary Table S1.

### 3.4. Myofibrillar Fragmentation Index

**Figure 4:**
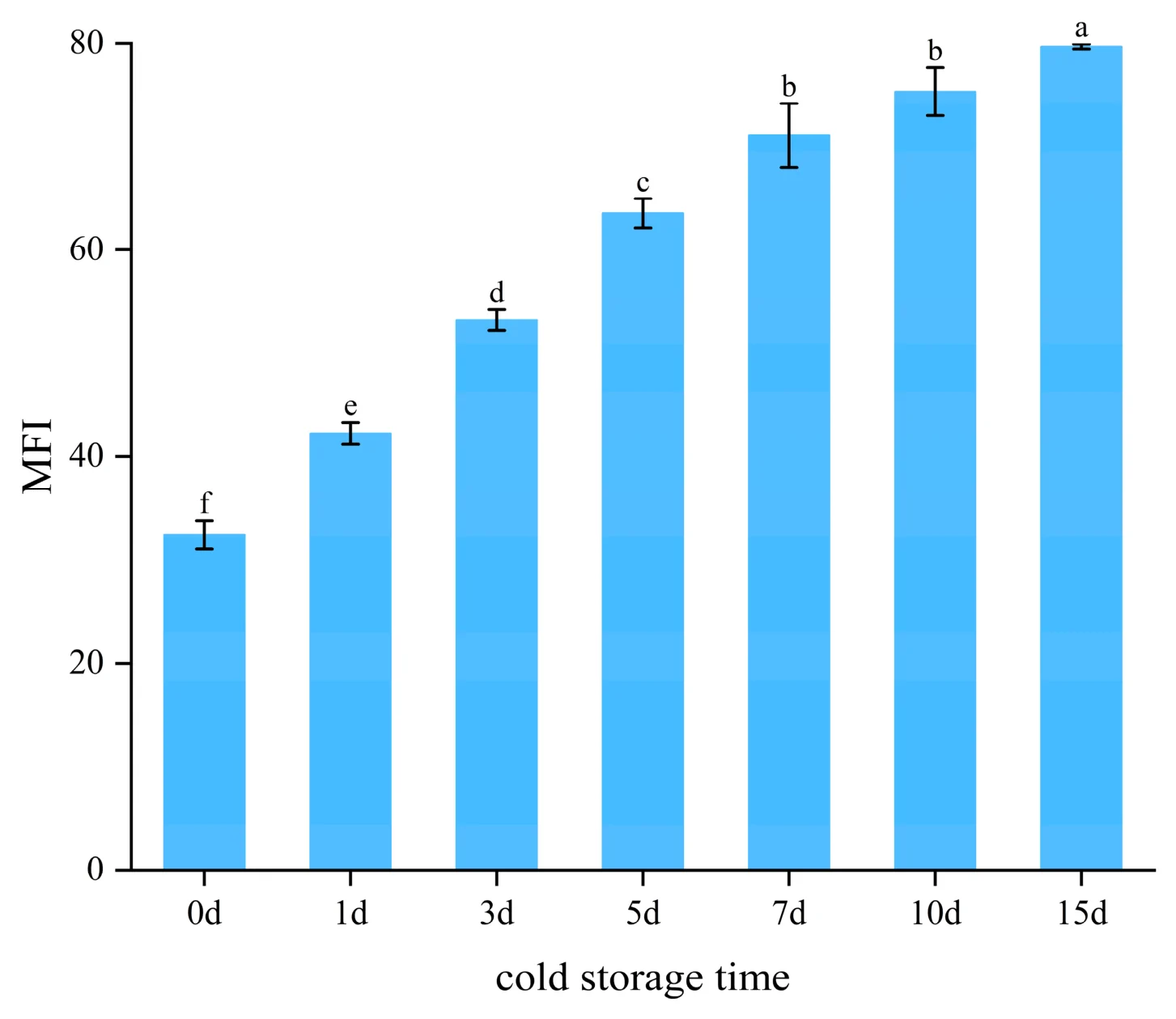
Changes in the mean fluorescence intensity (MFI) of *Esox lucius* muscle proteins during refrigerated storage at 4 °C for 0, 1, 3, 5, 7, 10, and 15 days. Data are presented as mean ± SD (*n* = 3 biologically independent samples per time point). Statistical analysis was performed using one-way ANOVA followed by Tukey’s post hoc test, with significant differences between time points denoted by different letters (*p* < 0.05). MFI, mean fluorescence intensity; SD, standard deviation; ANOVA, analysis of variance.

Myofibrillar fragmentation refers to the cleavage of myofibrils into smaller segments as a result of protein degradation. The MFI serves as a crucial indicator of the extent of protein degradation [27], and higher values signify greater disruption of the internal myofibrillar structure [28]. As shown in Figure 4, the MFI of northern pike exhibited a continuous upward trend during cold storage. Notably, the MFI increased rapidly from day 0 to day 7, rising from an initial value of 32.45 ± 1.36 to 71.10 ± 3.09, after which the rate of increase decelerated from day 7 to day 15, ultimately reaching 79.69 ± 0.24 on day 15. This pattern is consistent with the MFI changes observed in Coregonus peled (peled) stored at 4 °C by Guo et al. [29]. The observed increase may be attributed to the concerted action of endogenous and microbial proteases on myofibrillar backbone proteins, causing their degradation and fragmentation into smaller fragments, which in turn leads to a progressive rise in MFI with prolonged cold storage. The MFI trend demonstrates continuous degradation of myofibrillar proteins in northern pike during refrigerated storage.

### 3.5. TMT Quantitative Proteomics

#### 3.5.1. Protein Identification of *Esox lucius*

**Figure 5:**
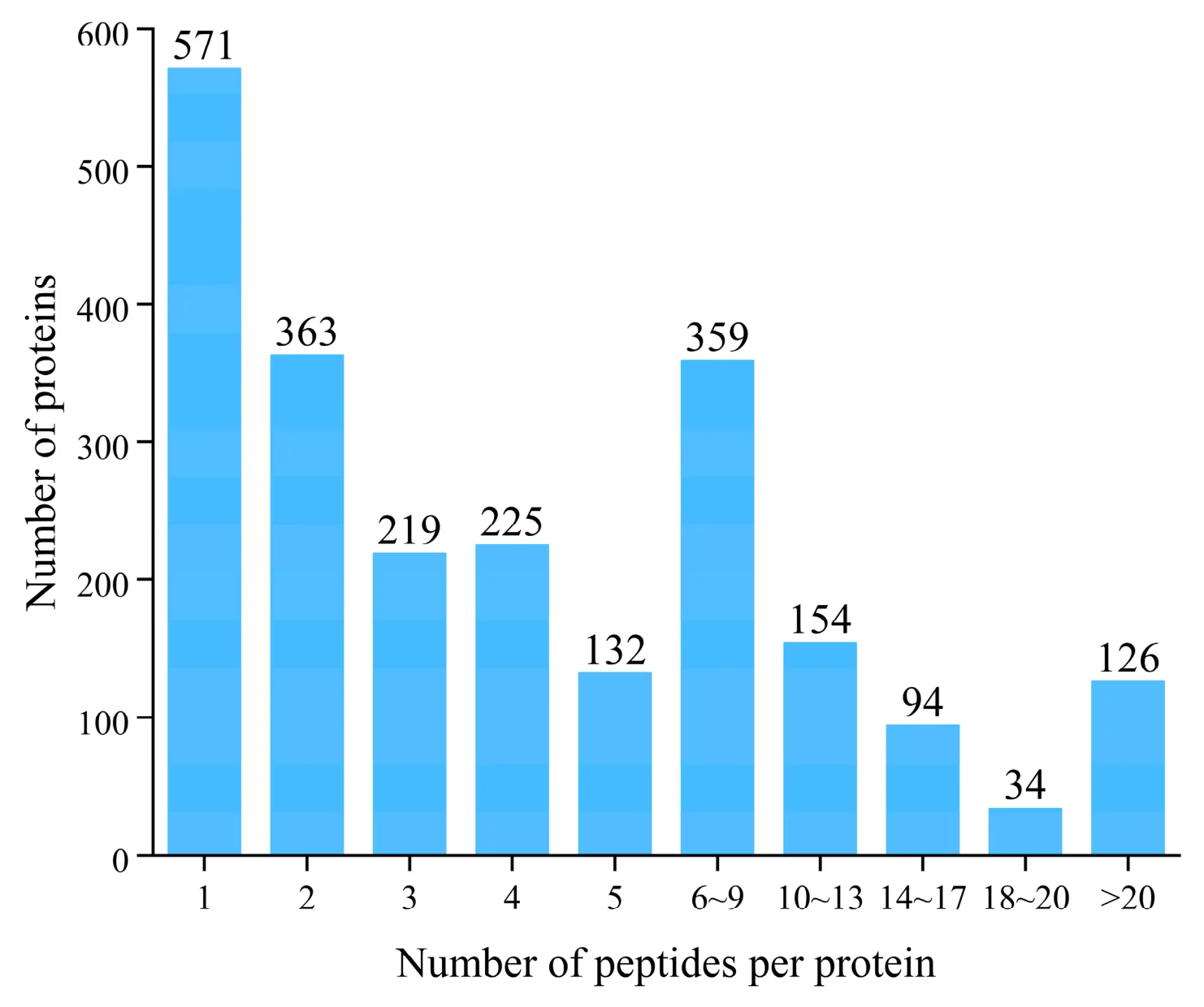
Distribution of peptide counts per identified protein. The horizontal axis represents the number of unique peptides per protein, and the vertical axis represents the number of proteins identified. Data were derived from TMT-based proteomic analysis of *Esox lucius* muscle samples.

**Figure 6:**
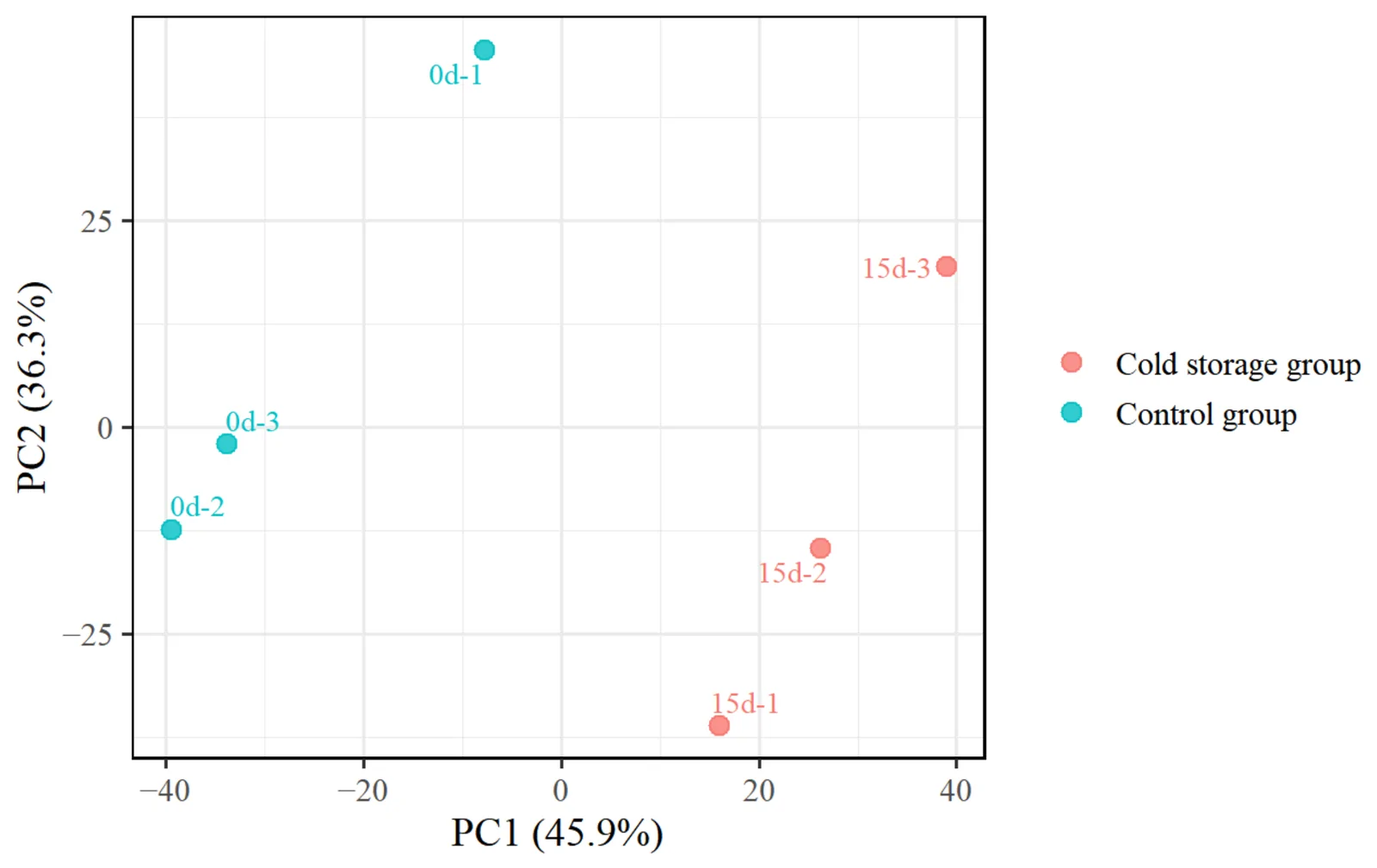
Principal component analysis (PCA) results of control and cold-stored groups of *Esox lucius*. The horizontal (PC1) and vertical (PC2) axes represent the first and second principal components, respectively, with the percentage of variance explained indicated in parentheses. Each dot represents an individual biological replicate, with different colors distinguishing between the control and cold-stored groups. PC1, principal component 1; PC2, principal component 2.

In this study, TMT-based quantitative proteomics was employed to analyze control and cold-stored northern pike (*Esox lucius*) samples, with three biological replicates for each group. A summary of the quality control metrics is provided in Supplementary Table S2. A total of 2277 confidently identified proteins and 15,514 peptides were identified. The distribution of peptide counts per identified protein is illustrated in Figure 5. Approximately 74.92% of the proteins contained at least two unique peptides, and approximately 94.47% comprised no more than 20 peptides, demonstrating the high reliability of the protein identification. The principal component analysis (PCA) results (Figure 6) revealed that, within a 95% confidence interval, the control and cold-stored groups were clearly separated, whereas samples within each group were clustered closely together [30]. This indicates that protein expression in northern pike undergoes pronounced changes under refrigerated conditions.

#### 3.5.2. Identification of Differentially Abundant Proteins

**Figure 7:**
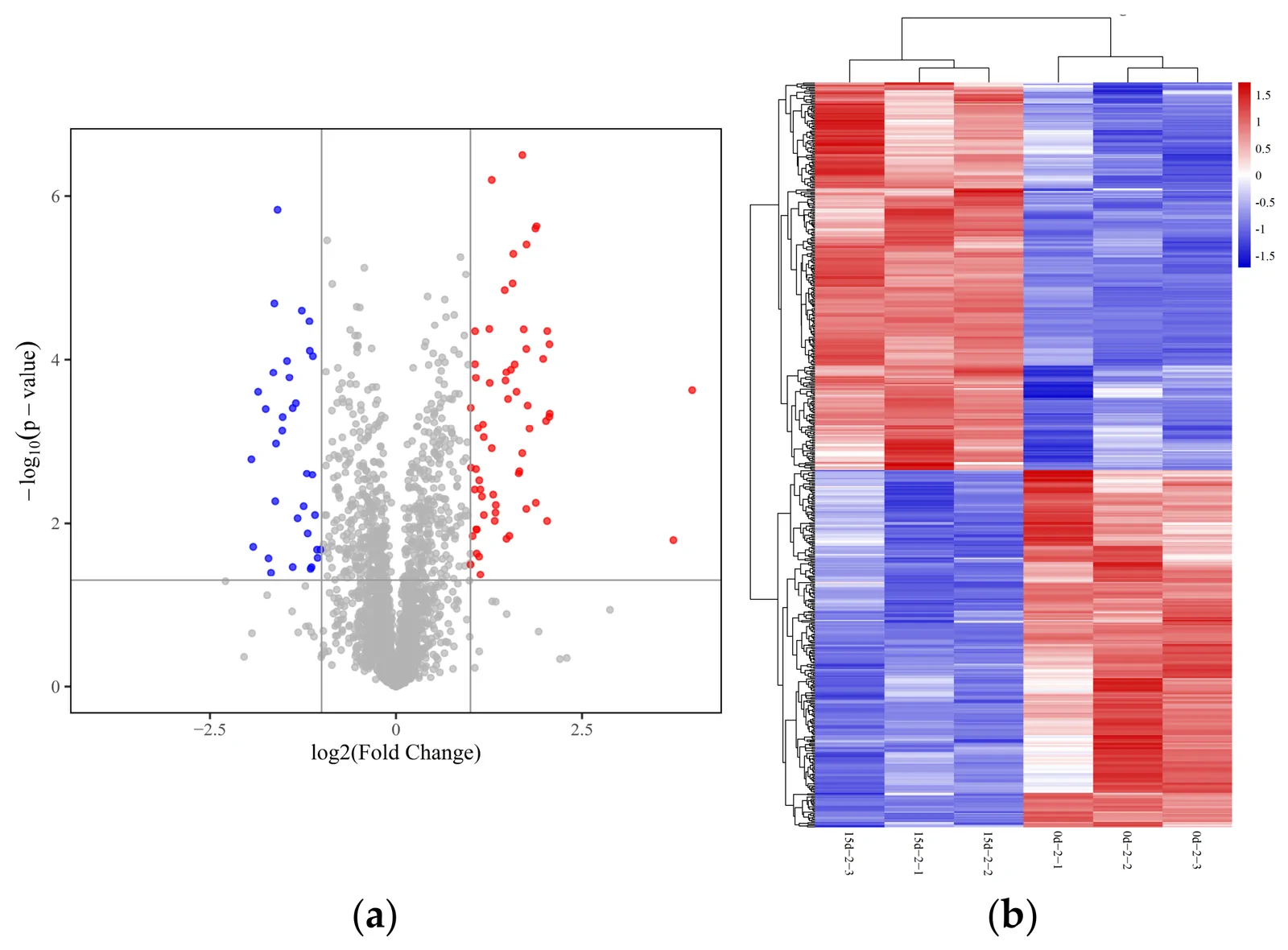
Analysis of differential protein expression between control and cold-stored groups of *Esox lucius*. (**a**) Volcano plot of differentially abundant proteins. The horizontal axis represents the log2-transformed fold change, and the vertical axis represents the −log10-transformed *p*-value. Red dots represent significantly upregulated proteins, blue dots represent significantly downregulated proteins, and gray dots represent proteins with no significant change. The significance threshold was set at *p* < 0.05 and fold change ≥1.2 or ≤0.83. (**b**) Hierarchical clustering heatmap of differentially abundant proteins. Each column represents an individual sample, and each row represents the differentially abundant proteins. Red and blue indicate high and low relative abundances, respectively. FC, fold change.

Proteins with high-confidence identification were retained based on a *p*-value < 0.05 and a fold change (FC) cutoff of ≤0.83 or ≥1.2. Compared with the control, 563 DAPs were detected in the refrigerated samples, of which 293 were upregulated and 270 were downregulated. Figure 7a shows a volcano plot illustrating the global DAP profile. Proteins with significant upregulation are marked in red, downregulated in blue, and those without significant changes are marked in gray. The hierarchical clustering heatmap in Figure 7b illustrates DAPs with distinct expression patterns using different color codes, where red indicates high abundance and blue indicates low abundance of DAPs. These results demonstrate that cold storage treatment markedly influences the protein expression profile of northern pike, and the abundance of a large number of proteins changes substantially.

#### 3.5.3. COG/KOG Analysis and Subcellular Localization

**Figure 8:**
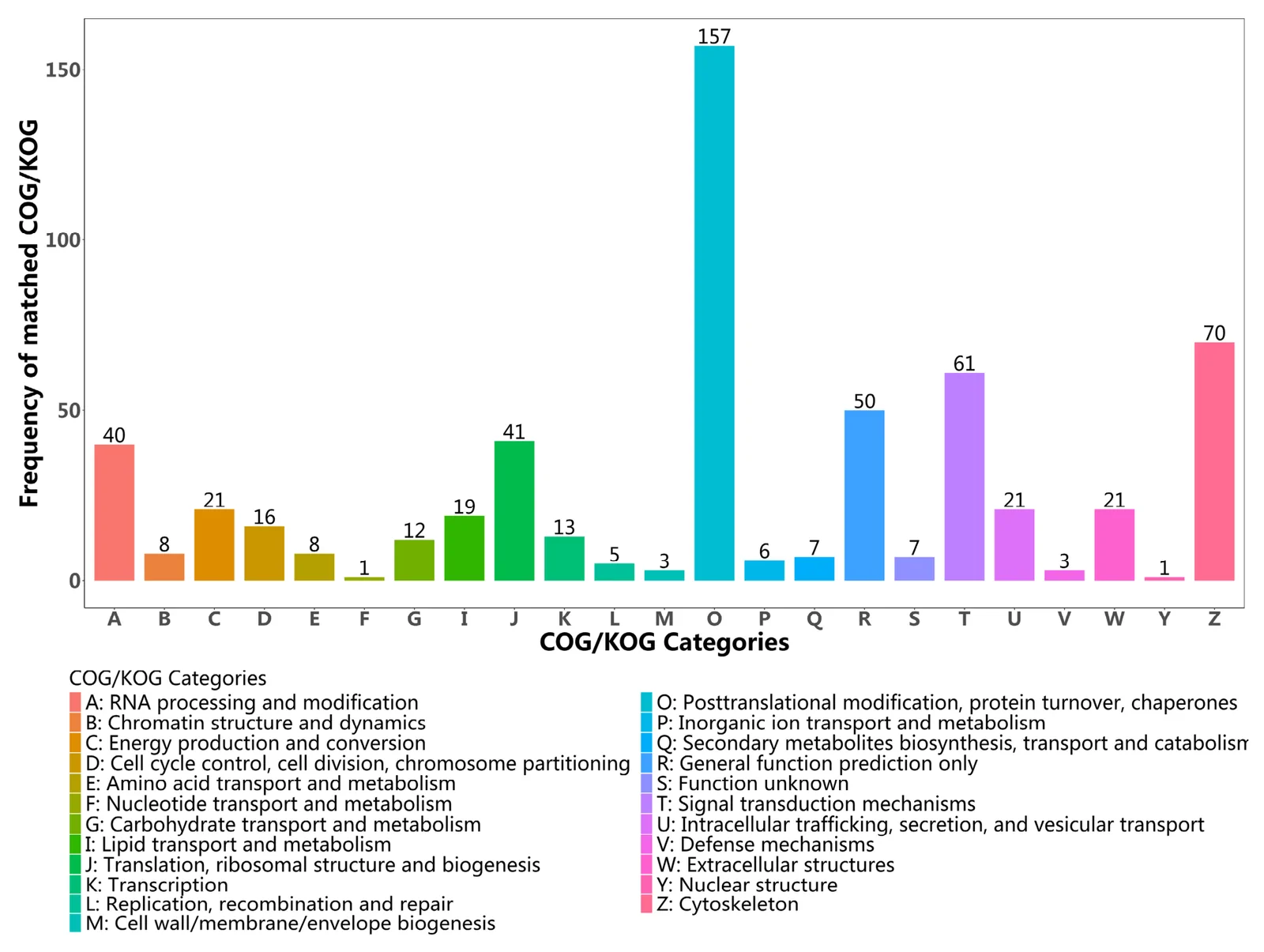
COG/KOG functional classification of differentially abundant proteins between control and cold-stored groups of *Esox lucius*. The horizontal and vertical axes represent the functional categories and the number of differentially abundant proteins annotated to each category, respectively. COG, Clusters of Orthologous Groups; KOG, Eukaryotic Orthologous Groups.

**Figure 9:**
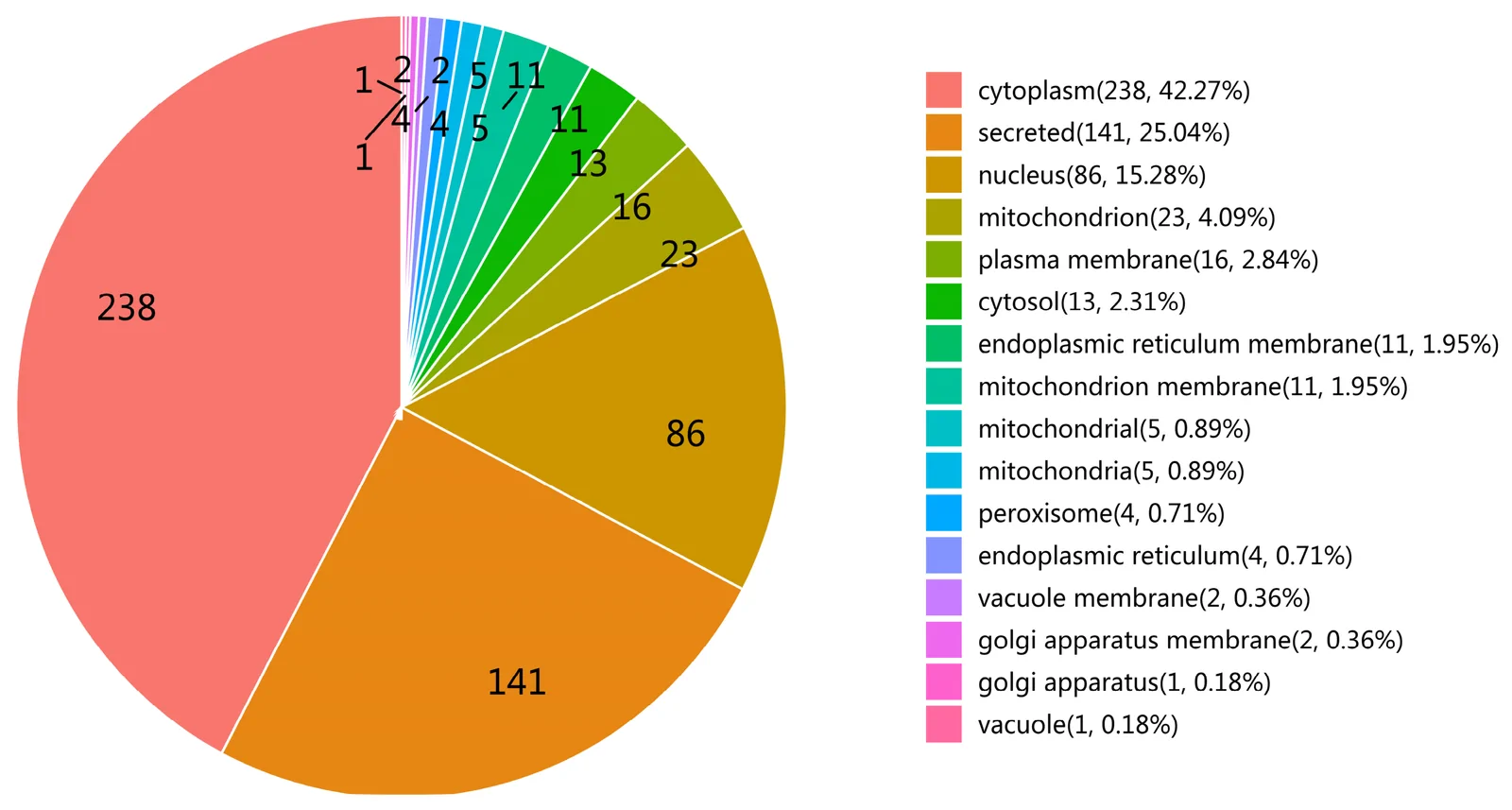
Subcellular localization analysis of differentially abundant proteins between control and cold-stored groups of *Esox lucius*. The pie chart shows the distribution of DAPs across different subcellular compartments, with each sector representing the percentage of proteins localized to a particular compartment. DAPs, differentially abundant proteins.

As shown in Figure 8, the differentially abundant proteins (DAPs) of northern pike under cold storage were assigned to 23 COG/KOG functional categories. The DAPs were primarily associated with the following COG functional categories: post-translational modification, protein turnover, and chaperones (O); cytoskeleton (Z); signal transduction mechanisms (T); translation, ribosomal structure, and biogenesis (J); and RNA processing and modification (A). Subcellular localization analysis (Figure 9) revealed that the DAPs were mainly distributed in the cytoplasm (42.27%), secretory vesicles (25.04%), nucleus (15.28%), and mitochondria (4.09%), indicating that cold storage-induced protein changes involve multiple subcellular compartments.

#### 3.5.4. GO Functional Enrichment Analysis

**Figure 10:**
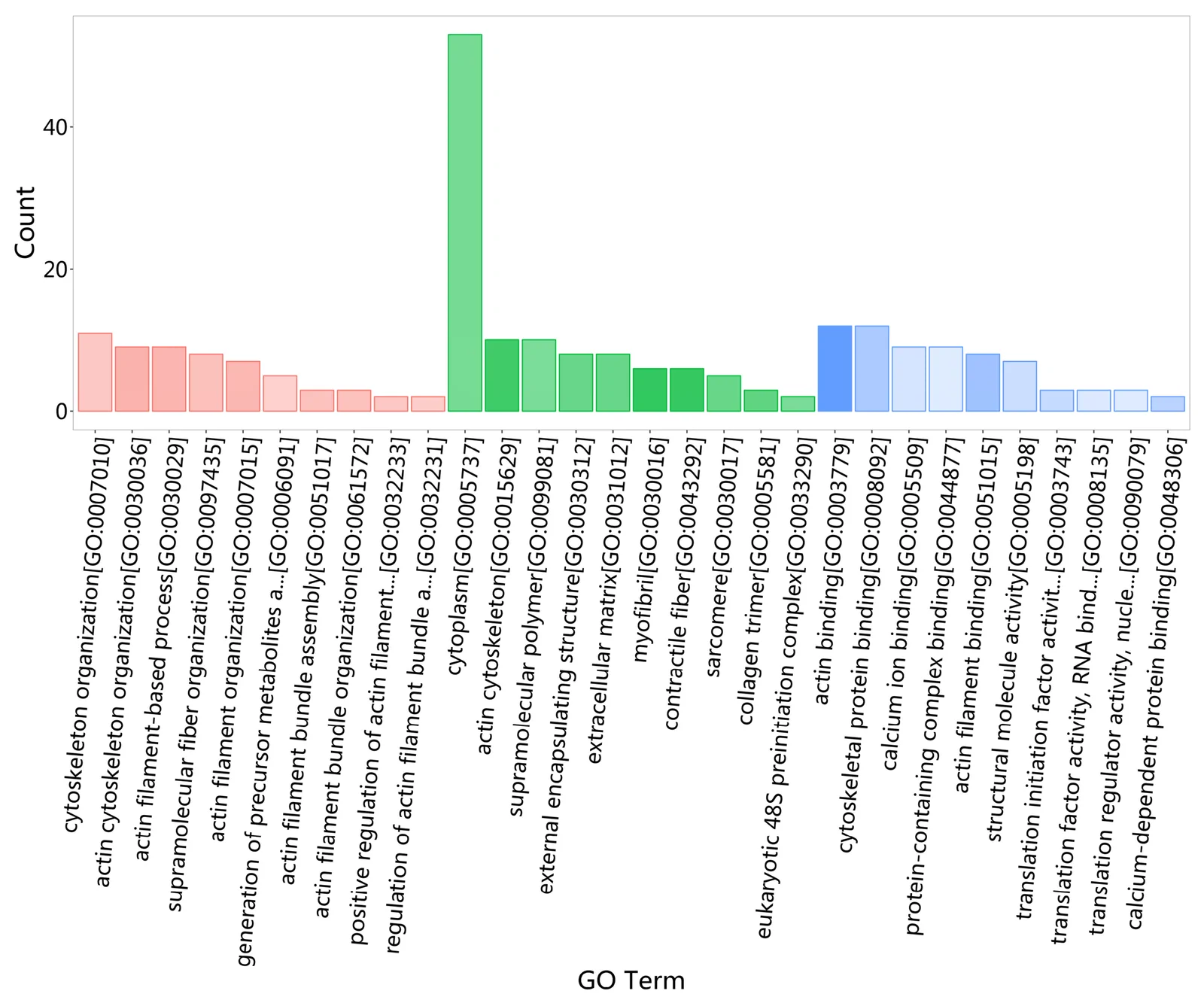
Gene Ontology (GO) enrichment analysis of differentially abundant proteins between control and cold-stored groups of *Esox lucius*. The horizontal axis represents the GO terms, and the vertical axis represents the number of differentially abundant proteins annotated to each term. The terms were categorized into biological process (BP, red), cellular component (CC, green), and molecular function (MF, blue). The transparency of each bar indicates the significance level (*p*-value), with darker colors representing lower *p*-values. GO, Gene Ontology; BP, biological process; CC, cellular component; MF, molecular function.

GO functional enrichment analysis was performed on the differentially abundant proteins (DAPs) between the control and cold-stored northern pike groups using the GO database. A complete list of the enriched GO terms is provided in Supplementary Table S3. The DAPs were categorized into three GO categories: biological process (BP), cellular component (CC), and molecular function (MF). As shown in Figure 10, within the BP category, DAPs were predominantly enriched in cytoskeleton organization, actin cytoskeleton organization, actin filament-based processes, and supramolecular fiber organization. In the CC category, DAPs were mainly enriched in the cytoplasm, actin cytoskeleton, supramolecular polymers, external encapsulating structures, and extracellular matrix. In the MF category, DAPs were primarily enriched in actin binding, cytoskeletal protein binding, calcium ion binding, protein-containing complex binding, and actin filament binding. These enrichment results were highly focused on the cytoskeletal system, and the BP, CC, and MF categories mutually corroborated each other, collectively indicating the organizational regulation and binding functions of the actin cytoskeleton. Within the CC category, 53 DAPs were enriched in the cytoplasm, reflecting a broad subcellular distribution of DAPs during cold storage, whereas the number of DAPs in other terms did not exceed 20, indicating that the functional alterations were relatively specifically concentrated in the cytoskeleton and its associated processes. To further pinpoint the core functional proteins, a correlation analysis between the DAPs and the physicochemical indicators was performed.

### 3.6. Correlation Analysis Between Differentially Abundant Proteins and Physicochemical Indicators

**Figure 11:**
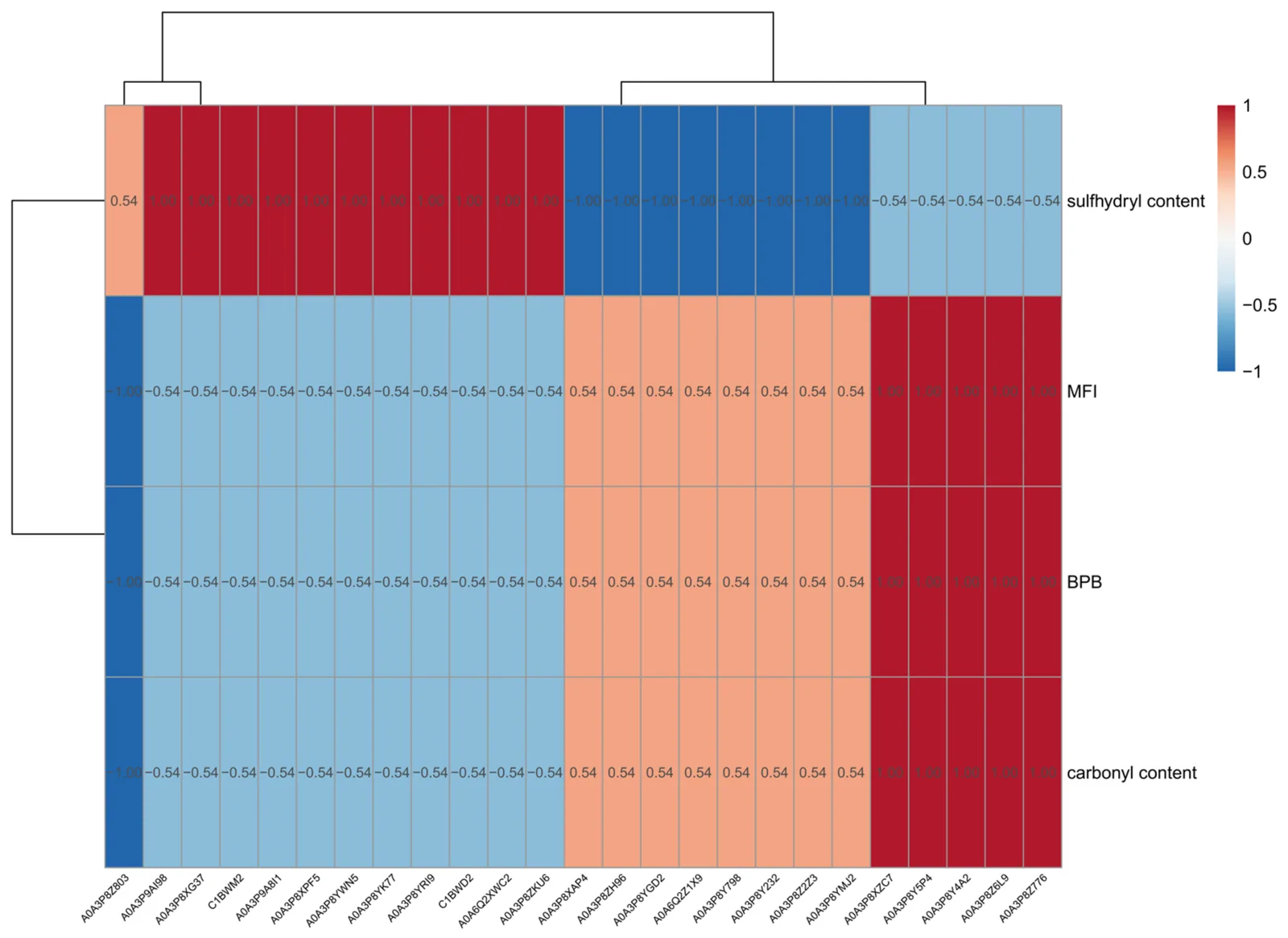
Cluster heatmap of correlation analysis between differentially abundant proteins and physicochemical indicators in *Esox lucius*. Note: Values in the heatmap represent Spearman correlation coefficients. The discrete values (−1, −0.54, 0.54, 1) arise from the limited sample size (*n* = 6) and pronounced between-group differences in the original data. Furthermore, given the limited sample size, this analysis is subject to inherent limitations.

As shown in Figure 11, twenty-five DAPs that satisfied the criteria were identified. The number of DAPs displaying significant strong correlations with surface hydrophobicity, carbonyl content, and MFI were each six, whereas 19 DAPs were significantly and strongly correlated with sulfhydryl content. Further screening revealed that six DAPs were significantly and strongly correlated with two or more physicochemical indicators: fructose-bisphosphate aldolase encoded by ALDOA (A0A3P8Z6L9), Nesh-SH3 domain-target protein (A0A3P8Z776), keratin 4 (A0A3P8Y4A2), neurofilament heavy polypeptide (A0A3P8Y5P4), serine/arginine repetitive matrix protein 2-like (A0A3P8Z803), and asymmetrical bis(5′-nucleosidyl) tetraphosphatase (A0A3P8XZC7).

## 4. Discussion

In this study, we systematically investigated the quality deterioration mechanisms of northern pike (*Esox lucius*) under refrigerated storage at the protein level by measuring physicochemical indicators, including surface hydrophobicity, carbonyl content, sulfhydryl content, and myofibrillar fragmentation index (MFI), combined with tandem mass tag (TMT)-based quantitative proteomics. The results demonstrated that with the extension of cold storage, surface hydrophobicity, carbonyl content, and MFI increased continuously, whereas sulfhydryl content declined persistently, indicating that the muscle proteins underwent marked oxidation and degradation. These changes are closely associated with the disruption of protein spatial structures, accumulation of oxidation products, and degradation of myofibrillar backbone proteins [31].

To further elucidate the protein-level characteristics of quality deterioration in northern pike between fresh and cold-stored states, TMT-based quantitative proteomics was employed to compare the muscle proteomes of fish stored for 0 and 15 d under refrigerated storage. A total of 2277 proteins were confidently identified, among which 563 were differentially abundant proteins (DAPs; 293 upregulated and 270 downregulated). Both COG/KOG functional and GO enrichment analyses consistently indicated that the functions of these DAPs were highly focused on the cytoskeletal system. In terms of biological processes, the DAPs were mainly involved in cytoskeleton organization and actin filament assembly. In terms of cellular components, they were predominantly localized to the cytoplasm, actin cytoskeleton and extracellular matrix. In terms of molecular functions, they were largely involved in actin binding, cytoskeletal protein binding, and calcium ion binding. These results demonstrate that the protein alterations induced by cold storage were primarily concentrated on the key structural components of muscle cells and that cytoskeleton-associated proteins underwent significant changes between the fresh and cold storage states. This finding is consistent with the observed disruption of myofibrillar structures and the progressive increase in MFI under refrigerated storage [32], suggesting that the loss of cytoskeletal integrity may be an important structural basis for the deterioration of northern pike muscle quality at the protein expression level.

Unlike previous fish proteomics studies that have primarily focused on the descriptive identification of differentially abundant proteins, this study integrated TMT-based quantitative proteomics with Spearman correlation analysis to establish associations between changes in protein abundance and multiple physicochemical indicators, thereby offering a more targeted strategy for screening candidate proteins associated with quality. Spearman’s rank correlation analysis between the 563 DAPs and the four physicochemical indices identified six proteins that displayed significant strong correlations with two or more indicators. It should be noted that the following discussion is based on correlational analyses and proposed as a working hypothesis rather than an established causal conclusion. These proteins played important roles in the changes in surface hydrophobicity, carbonyl content, sulfhydryl content, and MFI, and may serve as key factors underlying quality deterioration, as well as potential indicator proteins for monitoring quality changes in northern pike under refrigerated storage. Among them, fructose-bisphosphate aldolase functions as a glycolytic enzyme that catalyzes the reversible conversion of D-fructose-1,6-bisphosphate to dihydroxyacetone phosphate (DHAP) and D-glyceraldehyde-3-phosphate [33]. In this study, the abundance of this protein was significantly upregulated in the cold-stored samples compared to the fresh controls and exhibited significant positive correlations with surface hydrophobicity, carbonyl content, and MFI. One plausible interpretation is that the upregulation of fructose-bisphosphate aldolase may promote glycolysis, leading to the production of acidic metabolites such as lactic acid and a consequent decrease in muscle pH. An acidic environment can induce the denaturation and unfolding of myofibrillar proteins, exposing hydrophobic regions and thereby increasing surface hydrophobicity. Additionally, it can accelerate protein oxidation, resulting in an elevated carbonyl content. Moreover, acidic conditions may activate certain cathepsins, exacerbating the degradation of myofibrillar backbone proteins and ultimately contributing to an increase in the MFI [34]. However, these proposed mechanisms require further experimental validation. Keratin 4 and neurofilament heavy polypeptide are cytoskeletal proteins. Keratin, a major constituent of intermediate filaments, plays a protective role in maintaining cellular structural integrity and resisting external stress [35,36,37]. Both proteins were significantly upregulated in this study and positively correlated with MFI. It is hypothesized that following cold storage-induced damage to myofibrillar structures, the muscle cytoskeletal system attempts to upregulate these structural proteins for repair or stabilization; however, this stress-induced compensatory response remains insufficient to halt the progression of myofibrillar fragmentation, and thus MFI continues to rise. The Nesh-SH3 domain-target protein is involved in signal transduction and cytoskeletal reorganization. One plausible interpretation is that its upregulation correlated with increased surface hydrophobicity, carbonyl content, and MFI, suggesting that this protein may participate in cold-storage-induced muscle protein denaturation and degradation by modulating dynamic cytoskeletal rearrangements. However, the exact mechanism requires further experimental validation. Serine/arginine repetitive matrix protein 2-like has been implicated in RNA splicing regulation [38]. In this study, its expression was downregulated, and it may indirectly influence the expression of functional proteins related to northern pike quality by affecting the alternative splicing of pre-mRNAs; however, the precise mechanism requires further investigation. Asymmetrical bis(5′-nucleosidyl) tetraphosphatase belongs to the Nudix hydrolase family, participates in nucleotide metabolism and cellular homeostasis, and its core function is to catalyze the asymmetric hydrolysis of diadenosine tetraphosphate (Ap4A) [39]. This protein was significantly upregulated in the cold-stored samples compared to the fresh controls and displayed significant positive correlations with surface hydrophobicity, carbonyl content, and MFI. One plausible interpretation is that this enzyme may accelerate the breakdown of Ap4A, thereby reducing its intracellular accumulation in muscle cells. A decline in Ap4A levels may compromise the cellular capacity to counteract oxidative stress and low-temperature challenges, thereby indirectly accelerating the oxidation and degradation of myofibrillar proteins. However, this hypothesis requires further experimental validation.

Notably, the variation in sulfhydryl content was significantly correlated with 19 DAPs, far exceeding the number associated with the other three physicochemical indicators. This finding indicates that the conversion of sulfhydryl groups into disulfide linkages affects a wide range of proteins during refrigerated storage, representing a prominent manifestation of protein oxidation [40].

## 5. Conclusions

The results of this study demonstrated that with the extension of cold storage, the surface hydrophobicity and myofibrillar fragmentation index (MFI) of northern pike (*Esox lucius*) muscle exhibited continuous upward trends, while carbonyl and sulfhydryl contents displayed a highly significant negative correlation, indicating progressively intensifying protein oxidation. Using TMT-based quantitative proteomics to compare fresh samples (day 0) and samples after 15 days of refrigerated storage, 2277 proteins were identified. COG/KOG functional classification indicated that the differentially expressed proteins were significantly enriched in categories such as post-translational modification, protein turnover, chaperones, cytoskeleton, signal transduction, translation, ribosome structure and biogenesis, and RNA processing and modification. Further GO enrichment analysis demonstrated that these proteins mainly participated in biological processes, such as cytoskeletal organization and actin filament organization, were predominantly localized to intracellular components, and carried out molecular functions, such as actin binding and interactions with cytoskeletal proteins. Through correlation analysis, six proteins were identified as being significantly and strongly correlated with the physicochemical indicators under refrigerated storage: fructose-bisphosphate aldolase encoded by the ALDOA gene, Nesh-SH3 domain-target protein, keratin, neurofilament heavy polypeptide, serine/arginine repetitive matrix protein 2-like, and asymmetrical bis(5′-nucleosidyl) tetraphosphatase. These proteins may serve as candidate proteins of quality deterioration in northern pike during refrigerated storage. However, further experimental validation is required to confirm their utility. By leveraging proteomics techniques, this study provides insights into the protein alteration mechanisms underlying the quality deterioration of northern pike under refrigerated storage, based on a comparison between fresh and cold-stored endpoints, thereby providing a theoretical reference for the quality regulation of northern pike under refrigerated conditions and offering new insights into the development of cold storage preservation technologies for this species.

## Data Availability

The original contributions presented in this study are included in the article and Supplementary Materials. Further inquiries can be directed to the corresponding author.

## Supplementary Materials

The following supporting information can be downloaded at: https://www.mdpi.com/article/10.3390/foods15173088/s1, Table S1: Statistical Analysis Results; Table S2: QC; Table S3: GO Annotation Enrichment Analysis.
